# Supplementary material for: ‘You are too much in this modern world, that’s why you are like this’: Understanding perceptions of mental health among Somali women in London
Source: PLoS One. 2024 Oct 18;19(10):e0300299. doi: 10.1371/journal.pone.0300299 (PMC11488716; doi:10.1371/journal.pone.0300299)
Supplement: Attachment — Submitted filename: Gonzalez et al response to reviewers.docx [file pone.0300299.s001.docx]

‘You are too much in this modern world, that’s why you are like this’: Understanding perceptions of mental health among Somali people in London.

**Response to reviewers**

| **Comment: reviewer 1** | **Response** |
| --- | --- |
| Abstract: It needs to be revised to be a standard one. | The abstract we have included is based on the PLOS ONE submission guidelines. We are unsure how the reviewer would like us to change this. If it still needs changing please specify how you would like it to be changed. |
| Method: Why all women were selected for the research needs clarification | We have added a clarification in the text. |
| Method: What are the mental health conditions of these selected women? | None of the participants are suffering from mental health condition. The intention was to understand views on mental health among a group of community members, rather than those suffering from a mental health condition. |
| Method: Why is the sample only 7? | Recruitment of participants was challenging and it was impossible to recruit more people in the time available. Nonetheless, the interviews generated in-depth, rich descriptions of the phenomenon in question. |
| Method: I found from the result section that none of the women had/had mental health conditions, as they mostly referred to their relatives or friends. In such a context, interviews with 7 women must be justified as it is difficult to establish an argument based on a very small number of interviews. This can be a deciding factor if it is to be accepted or rejected by the editor. Thus, we need very clear and justified responses in the manuscript. | We did not interview people with a mental health condition because we wanted to get a general overview of people’s views about mental health, rather than their personal experiences of it.  In terms of the number of interviews, we are not claiming that the article is representative of the Somali women in London in general. The article uses qualitative methods and, as such, we are seeking rich descriptions of the perceptions and experiences of the people we interviewed, rather than trying to draw conclusions for a social group as a whole. We are also not seeking theoretical saturation as the study is not based on grounded theory and we are not seeking to generate theory from empirical data. We have added extra text outlining these points in the manuscript. |
| Result: Quotes: Please use a citation or (add some basic information about the respondent of the quote, i.e., 60 years old woman. | We have added this information. |
| Result: Organising the results into two or three subsections based on the key themes would make the section clear and easy for the reader. | We have organised the results section into three themes: Personal importance of mental health, Stigma and community judgement and Community diversity: geography and age. |
| Discussion: Participants in the research did not discuss traditional healers. However, the author discusses this issue, which is unrelated to the result. Instead, enriching the discussion around the spiritual issues and interpretations of mental illness would make the last part of the discussion interesting. | There was one paragraph that included references to traditional methods of treating mental illness. We have removed this paragraph. |

| **Reviewer 2: comments** | **Response** |
| --- | --- |
| Under data collection and sampling section of the manuscript, it is stated clearly that “this study recruited seven participants, who were all women”. However, the title is presented as perceptions of mental health among Somali people in London. Given the fact that this is a qualitative study, where voice is fundamental, it is advisable that the title adequately reflects the voices captured in the study. I recommended ‘Somali women in London’ instead of ‘Somali people in London’. | We have changed the title so it now reads ‘You are too much in this modern world, that’s why you are like this’: Understanding perceptions of mental health among Somali women in London.  We have also changed the wording in various places to reflect this change. |
| The sample of seven women should also be clearly included in the abstract for clarity. | We have added this information. |
| In the abstract, it is stated that ‘description-focused thematic analysis’ was used to interpret key themes. Under data analysis section of the methodology, it is stated that description-focused coping was done, and then thematic analysis was used to identify themes and patterns. This presents difficulty and inconsistency regarding what constitutes thematic analysis and description-focused thematic analysis. | We have changed the wording in the data analysis section so it now reads ‘description-focused thematic analysis’. This provides a more accurate description of what was done. |
| The data analysis as presented in the methodology is scanty and does not present enough information to convince readers of the rigor of the analysis. | We have added more detail in the text. |
| Respondents and participants are interchangeably used in this manuscript. There is the need to stick to interpretivist language and terminologies through out the document. | We have changed all instances of ‘respondent’ to ‘participant’. |
| Under the research paradigm and question, it is stated that “The main goal of the article is to analyze the meanings people attach to mental health based on their experiences”. More context is needed to understand what is meant by ‘their experiences’. What experiences are being referred to here? Lived experiences? | We have removed the words ‘based on their experiences’ as we felt that this was misleading. |
| In the last statement of the concluding paragraph in the same section, it is stated that “This research is therefore intended as a sample of the views of Somali people living in London, where the opinions and views expressed in this article are developing and changing.” The paper is exploring perceptions of mental health. So what does it mean to say opinions and views expressed are ‘developing’ and ‘changing’? | We have changed the wording of this section so it reads ‘This research is therefore intended as a sample of the views of Somali women living in London at a particular point in time.’ |
| Under the results section, the first statement reads “This research aimed to gather better understanding of the perspectives of mental health among Somali people living in London.” What is the meaning and significance of ‘better understanding’ in this context? | We have changed the wording of this section so it reads ‘This research aims to understand the perspectives on mental health of a group of Somali women living in London’. |
| The manuscript needs major revisions in ways that present philosophical, methodological, and interpretive clarity. In its current state, it is difficult to understand the significant contributions that this manuscript makes to current knowledge, partly because of these inconsistencies. | We have made significant changes to various elements of the manuscript to address this comment. We hope that these changes make our contribution clearer. |
